# Exploring the Agromorphological Profiles of the Cacao (*Theobroma cacao* L.) Collection from the INIA Germplasm Bank in the Amazonas Region, Peru

**DOI:** 10.3390/plants14223536

**Published:** 2025-11-19

**Authors:** José Jesús Tejada-Alvarado, Nuri Carito Vilca-Valqui, Luis Alberto Montenegro-Acuña, Jhimy Andy Parco-Quinchori, Elizabeth Fernandez

**Affiliations:** 1 Estación Experimental Agraria Amazonas, Dirección de Recursos Genéticos y Biotecnología, Instituto Nacional de Innovación Agraria (INIA), km 3.5 (Carretera Aeropuerto), Chachapoyas 01001, Peru; montenegro9824@gmail.com; 2 Centro Experimental La Molina, Dirección de Recursos Genéticos y Biotecnología, Instituto Nacional de Innovación Agraria (INIA), Av. La Molina 1981, Lima 15024, Peru; jhmparco@gmail.com (J.A.P.-Q.); efernandezh@inia.gob.pe (E.F.)

**Keywords:** characterization, conservation, descriptors, FTIR, HPLC, plant genetic resources, theobromine

## Abstract

Cacao is a strategic crop in Peru due to its significant socioeconomic impact, driving extensive efforts to collect, characterize, and conserve its genetic diversity. This study aimed to establish phenotypic criteria to differentiate and structure the Cacao Amazonas Perú (CAP) germplasm, thereby providing a foundation for selection and breeding programs. A total of 113 accessions from the INIA Germplasm Bank were evaluated over two consecutive growing seasons using 33 quantitative and 18 qualitative agromorphological descriptors. Data were analyzed through univariate and multivariate approaches. The results revealed substantial phenotypic variability, with coefficients of variation reaching up to 37.5% for fruit-related quantitative traits, all exhibiting high heritability values (>60%). Principal component analysis indicated that the first two components explained 29.3% of the total variance, primarily influenced by fruit and seed descriptors. Hierarchical clustering analysis identified eight phenotypic groups; one cluster exhibited high seed mass and a favorable pod index (17.63), while another showed the highest seed index (1.55 g) and the greatest intragroup distance (7.54). This comprehensive characterization highlights accessions with superior agronomic and bioactive potential, providing a robust framework for parental selection, core collection development, and targeted breeding strategies to enhance cacao competitiveness and resilience under changing climatic conditions.

## 1. Introduction

Climate change and demographic pressure pose critical challenges for agriculture in tropical regions, where limited availability of water and/or nutrients may constrain the advances in crop improvement [1]. In response, the development of cultivars resilient to fluctuating environmental conditions has emerged as a priority strategy to ensure sustainable productivity and reinforce food security. Within this framework, the systematic conservation and characterization of genetic diversity acquire scientific significance, as germplasm represents a strategic reservoir of adaptive alleles [2].

The chocolate tree (*Theobroma cacao* L.) is a tropical cauliflorous species of the Malvaceae family [3], domesticated approximately 3500 years BC in northwestern South America [4,5]. This chronology is supported by fossil evidence recovered from archaeological sites of pre-Columbian cultures such as Chinchipe (Ecuador) and Montegrande (Peru) [6]. Cocoa, a strategic raw material for the chocolate industry, represents a major global economic asset owing to its high added value derived from a biochemical composition rich in secondary metabolites and alkaloids with immunomodulatory properties [7,8]. Its economic relevance is further reflected in projections for the global chocolate market, estimated at USD 142.88 billion in 2024, with a sustained annual growth rate of 3.5%, expected to reach approximately USD 194.73 billion by 2033 [9].

Globally, three main cacao groups have traditionally been recognized: Criollo, Forastero, and their hybrid Trinitario [10]. However, advances in phylogenomic analysis have enabled the reclassification of cacao diversity into eleven distinct genetic groups: (I) Amelonado, (II) Contamana, (III) Criollo, (IV) Curaray, (V) Guayana, (VI) Iquitos, (VII) Marañón, (VIII) Nacional, (IX) Nanay, (X) Purús, and (XI) Nacional Boliviano [11,12]. These groups are distributed throughout the neotropical regions of South and Central America, representing a gradient of domestication and bioclimatic adaptation. Within this geographic context, Peru emerges as a hotspot of genetic diversity, harboring seven of the eleven identified groups (Contamana, Curaray, Iquitos, Marañón, Nanay, Purús, and Nacional), thereby positioning the country as a reservoir of wild alleles and agronomically valuable variants [12,13].

In Peru, cacao cultivation extends across the mid and high-altitude jungle ecoregions, ranging from Cusco to Amazonas [6]. Among these, the Amazonas region is distinguished by the production of a wide diversity of cacao beans, which are renowned for their appealing sensory attributes and have earned the region the protected designation of origin Cacao Amazonas Perú (CAP) [14]. This distinction has facilitated the capitalization of growing market demand, substantially enhancing the international competitiveness of the product, considering that fine-flavor cocoa represents approximately 75% of Peru’s total cocoa exports [15].

In the Amazonas region, the Nacional and Criollo varieties are the principal representatives of fine-flavor cacao [16]. Criollo is cultivated across 22.5% of the production area in the province of Bagua, 72.7% in Utcubamba, and 78.3% in Condorcanqui [17]. Owing to its wide distribution, cacao cultivation makes a significant contribution to regional development, benefiting broad sectors of the population, particularly those in vulnerable socioeconomic conditions.

To maximize crop profitability, farmers prioritize the selection of genotypes that exhibit desirable traits such as resilience to biotic and abiotic stresses, high fruit productivity with morphologically appealing pods, and large seeds possessing distinctive organoleptic profiles [18]. However, both intra- and inter-population genetic variability are known to fluctuate spatiotemporally under the influence of reproductive, geographical, and anthropogenic factors, thereby contributing to phenotypic heterogeneity [19]. Under these circumstances, the systematic characterization of a wide range of accessions becomes essential for elucidating advantageous gene combinations and identifying promising plant genetic resources [20].

Phenotypic agrobiodiversity is conventionally assessed through the evaluation of qualitative and quantitative traits [2]. Among these, average seed weight stands out as a key quantitative descriptor due to its high heritability. Seed size and morphometric uniformity are regarded as priority attributes in the chocolate industry, thereby justifying their inclusion as a fundamental criteria in the design of assisted genetic selection programs [18,21]. This perspective underscores the methodological relevance of morphological characterization as an analytical foundation for population genetics studies, while remaining accessible and farmer-friendly [21,22]. Consequently, deepening the understanding of which phenotypic traits discriminate the Amazonian germplasm will enable the identification and selection of elite accessions for crop improvement.

Numerous morphological descriptors have been documented in the literature for the characterization of commercially important crops, including *Coffea arabica* L. [23], *Amaranthus* [24], *Hibiscus sabdariffa* L. [25], *Phoenix dactylifera* L. [26], and *Capsicum* spp. [27], among others. In the case of cacao, 65 agromorphological descriptors were established in the late twentieth century and have since been systematically adopted by international organizations such as the Tropical Agricultural Research and Higher Education Center (CATIE), the International Cocoa Genebank Trinidad (ICGT), and the International Cocoa Germplasm Database (ICGD), which have supported comparative studies and genomic conservation efforts for decades [28]. Building on this framework, a recent study conducted in northeastern Peru [6], identified five groups within a population of 146 fine-flavor cacao ecotypes, classified as Toribianos, Utkus, Cajas, Indes, and Bagüinos. Among these, the latter two groups are notable for their adaptation to elevations above 500 m, exhibiting superior organoleptic profiles and high productivity, as evidenced by an improved pod index (<13.88).

In light of Peru’s recognized richness in plant genetic resources, this study explicitly addresses the existing knowledge gap regarding the definition of phenotypic criteria for differentiating and structuring of the CAP germplasm. To this end, we systematically evaluated 113 accessions from the INIA Germplasm Bank using an integrated set of 51 standardized descriptors and implemented a multivariate analytical framework aimed at identifying discriminant traits and delimiting promising accessions with notable phytochemical potential. This approach seeks to generate robust and operational evidence to strengthen the position of Peruvian cacao and guide its selection, conservation, and genetic improvement under the current context of climate change.

## 2. Materials and Methods

### 2.1. Germplasm Collection

**Figure 1:**
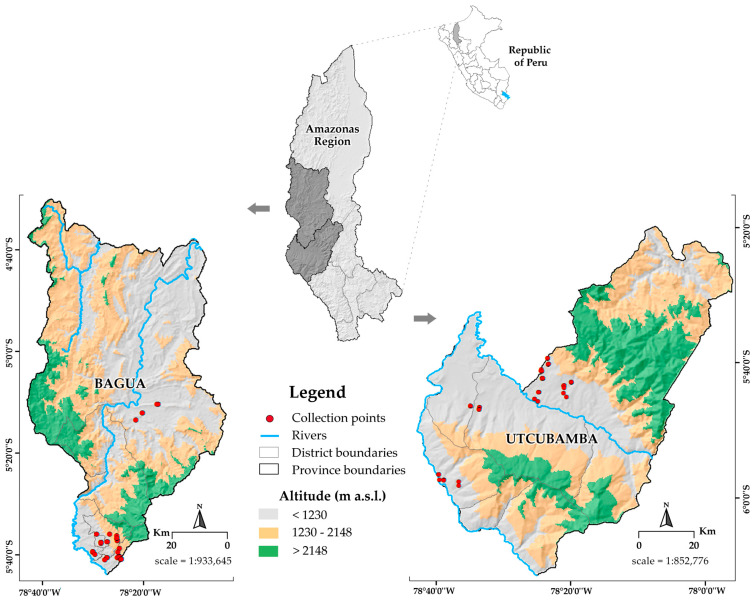
Cartographic collection of the 113 cacao accessions collected from the Amazonas region, Peru. The collection sites are shown for two provinces (Bagua and Utcubamba) using shared symbology but different map scales. The projection is based on the World Geodetic System 1984 (WGS 84), Universal Transverse Mercator (UTM) Zone 17S, Datum WGS84.

During 2016, plant material (scions) was collected from the middle third of the canopy of previously identified mother trees located in natural populations within the provinces of Bagua and Utcubamba (Figure 1). The selection of these specimens was carried out strategically, based on distinctive morphological characteristics recognized by local producers. This process allowed for the recording of passport data and the subsequent assignment of a unique “PER” code (Table S1), which identifies each accession conserved in the Germplasm Bank of the National Institute of Agrarian Innovation (INIA-Peru) [29].

The study provinces, located in Amazonas region, are characterized by pronounced geographic, climatic, and altitudinal variability, as previously documented by Vásquez-García [30]. This environmental heterogeneity creates an ecological mosaic that fosters a broad expression of phenotypic variability among the evaluated cacao accessions.

**Figure 2:**
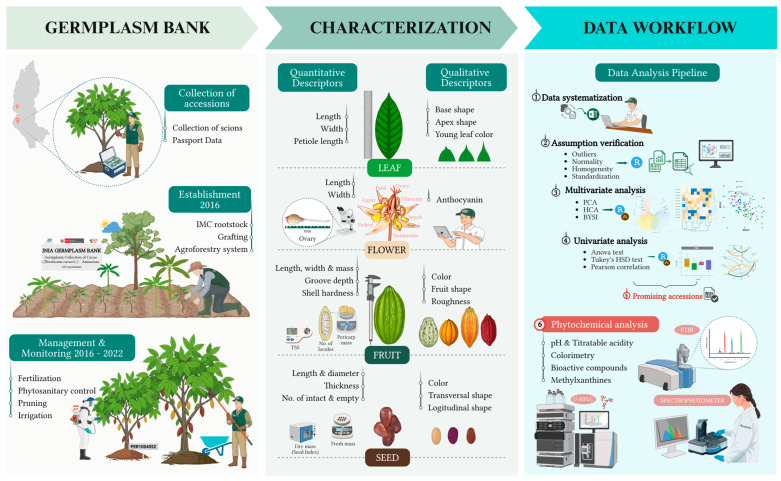
Schematic flow of the methodological framework applied in the study.

The germplasm collection, consisting of buds from 113 accessions, was grafted onto the universal rootstock IMC 67, known for its vegetative vigor and disease resistance. The rootstocks were previously cultivated under shade house conditions to ensure uniform and pathogen-free growth. The establishment was carried out through top cleft grafting during the same year of collection, using scions with a diameter of 10 mm on rootstocks transplanted to the final field (Figure 2). The grafts were installed within an agroforestry system following a Latin square experimental design, with a spacing of 3 × 3 m^2^. Each accession comprised nine grafts, individually identified with QR codes to ensure traceability. During the first two years, the system was intercropped with *Musa paradisiaca* L., while the forest species *Cedrela odorata* L., *Inga edulis* Mart., and *Calycophyllum spruceanum* Benth., planted at 10 m intervals, reached their developmental stage.

#### 2.1.1. Location

The *ex situ* conservation of the cacao collection maintained by the Germplasm Bank of INIA–Peru was established at the Huarangopampa Experimental Center (EC), a subsidiary of the Amazonas Agrarian Experimental Station. This site is located in the district of El Milagro, Utcubamba province (5°39′50″ S, 78°32′01″ W), at an altitude of 480 m a.s.l., where the collection comprises a total of 1017 plants distributed across 1 ha (Figure S1).

#### 2.1.2. Agronomic Management

During the establishment and maintenance phases, standardized cultural practices were implemented, including weed control, periodic irrigation according to plant water requirements, and split fertilization applied twice per season. The latter was determined based on the crop’s nutritional demands, as established through systematic soil analyses conducted throughout the evaluation period.

In addition, formative and maintenance pruning were consistently performed over six consecutive years, accompanied by continuous phytosanitary monitoring to ensure the timely detection of pests and diseases (Figure 2). During this period, graft viability was confirmed, and most accessions reached a stable productive stage. From this point onward, the evaluation of agromorphological traits was scheduled when the plants reached seven years of age (2023 season) and eight years (2024 season), encompassing two consecutive evaluation cycles.

#### 2.1.3. Agroecological Conditions

The initial edaphic conditions of the site were characterized by a clay-texture soil, with a pH of 7.8 and an electrical conductivity of 9.9 mS/m. Regarding mineral availability, the soil analysis indicated concentrations of 3.9 mg/kg of phosphorus, 176.6 mg/kg of potassium, 7.2% total carbon, 0.7% organic matter, and 0.4 mg/g total nitrogen. Furthermore, the concentration of exchangeable bases was 26.5 cmol(+)/kg for calcium, 3.0 cmol(+)/kg for magnesium, and 0.6 cmol(+)/kg for sodium. These properties were determined through analytical characterization conducted by the Soil, Water, and Foliar Analysis Laboratory (LABSAF) of INIA, which is accredited according to the NTP-ISO/IEC 17025:2017 standard [31] under registration No. LE-200.

During the characterization period, the climatic conditions in the collection area were characterized by a higher accumulated precipitation in 2023 (1040.9 mm) compared with 2024 (926.5 mm) [32]. Temperature remained relatively stable across both years (Figure S2), whereas relative humidity exhibited a marked decline between July and September in both periods, coinciding with the months of lowest rainfall.

### 2.2. Agromorphological Characterization

A total of 51 phenotypic descriptors were employed for the agromorphological characterization of the germplasm [33,34,35], comprising 33 quantitative and 18 qualitative traits. The selection of these descriptors was based on their capacity to reveal the distinctive characteristics of each accession, encompassing key morphological structures such as leaves, flowers, fruits, and seeds (Figure 2).

#### 2.2.1. Quantitative Descriptors

Leaf: LL = Leaf length (cm), LW = Leaf width (cm), PtL = Petiole length (cm).

Flower: PdL = Pedicel length (cm), SpL = Sepal length (mm), SW = Sepal width (mm), PL = Petal length (mm), PW = Petal width (mm), LgW = Ligule width (mm), FL = Filament length (mm), StL = Staminode length (mm), SL = Style length (mm), OL = Ovary length (mm), OW = Ovary width (mm).

Fruit: SH = Shell hardness (MPa), FM = Fruit mass (g), FrL = Fruit length (cm), FW = Fruit width (cm), PrT = Pericarp thickness (cm), GD = Groove depth (cm), PM = Pericarp mass (g), NL = Number of locules (unit), TSS = Total soluble solids (°Brix).

Seed: NSL = Number of seeds per locule (unit), FSMF = Fresh seed mass per fruit (g), NSF = Number of seeds per fruit (unit), NIS = Number of intact seeds (unit), NES = Number of empty seeds (unit), SeL = Seed length (mm), SD = Seed diameter (mm), ST = Seed thickness (mm), SI = Seed index (g) and PI = Pod index (unit). The mathematical equations used to calculate SI and PI are provided below [36]:

(1) $$SI=\frac{Dry\;mass\;of\;n\;seeds\;(g)}{n}$$

(2) $$PI=\frac{1000\;(g)}{\left[Average\;seed\;number\right]\times [Average\;mass\;of\;dry\;seed\;(g)]\;}$$

#### 2.2.2. Qualitative Descriptors

The qualitative morphological descriptors are detailed in Table 1.

### 2.3. Phytochemical Characterization

Fresh cotyledons from the promising accessions, together with a representative accession to ensure the inclusion of all phenotypic groups in the analysis, were preserved at −80 °C for 24 h and subsequently lyophilized under a pressure of 0.003 mbar for 72 h (Labconco, Corp., Kansas City, MO, USA, −84 °C). The resulting samples were ground, sieved (850 μm), and defatted following the protocol adapted from Cortez [37]. The extract was filtered with Whatman paper No. 40 and stored in amber Eppendorf-type tubes at −22 °C. All analyses were performed in triplicate.

#### 2.3.1. Colorimetric Measurement, Titratable Acidity, and pH

The color of the lyophilized powder was determined in the CIELAB color space (L*, a*, b*), using a CR-400/DP-400 digital colorimeter (Konica Minolta Inc., Tokyo, Japan). Simultaneously, 10 g of sample were dissolved in 50 mL of Milli-Q water at 100 °C. The mixture was briefly vortexed and filtered using Whatman No. 40 paper. The pH of the solution was measured using an HI2211 pH meter (Hanna Instruments, Woonsocket, RI, USA). Titratable acidity (TA) was determined by titration to pH 8.1 using 0.1 N NaOH, incorporating three drops of phenolphthalein as an indicator. The results were expressed as a percentage of acetic acid equivalent.

#### 2.3.2. Bioactive Compound Profile

The antioxidant capacity was evaluated using the DPPH free radical scavenging assay, following the procedure described by Brand-Williams [38], with minor modifications. For this purpose, 100 μL of the extract was mixed with 3.9 mL of DPPH solution in amber Eppendorf tubes, and the absorbance was measured at 515 nm using a UV/Vis spectrophotometer (Genesys 180, Thermo Scientific™, Madison, WI, USA). Quantification was carried out using Trolox standards (y = 0.2321x − 1.2859; R^2^ = 0.9978), and the results were expressed as milligrams of Trolox equivalents (TE) per gram of sample (mg TE/g).

Total phenolic content (TPC) was determined using the Folin–Ciocalteu reagent [39]. For this, 500 μL of the extract was combined with 2.5 mL of 10% reagent and 2 mL of Na_2_CO_3_ (4% *w*/*v*) in amber tubes. The absorbance was then measured at 750 nm using a UV/Vis spectrophotometer. Concentrations were calculated from a gallic acid calibration curve (y = 0.0036x + 0.1872; R^2^ = 0.9982), and the results were expressed as milligrams of gallic acid equivalents (GAE) per gram of sample (mg GAE/g).

#### 2.3.3. HPLC Screening of Methylxanthines and Phenolic Compounds

Individual standard solutions of theobromine, caffeine, caffeic acid, catechin, epicatechin, and cyanidin 3-O-glucoside were prepared, with concentration ranges and retention times established according to the optimized protocol described by Cortez [40]. The analysis was performed using an ultra-high-performance liquid chromatograph (Agilent 1290 Infinity II, Agilent Technologies, Santa Clara, CA, USA) equipped with a Zorbax Eclipse Plus C18 column (4.6 × 25 cm^2^, 5 μm) coupled to an automated diode-array detector (DAD). The operational conditions included an injection volume of 20 μL, a total run time of 22 min, and a constant temperature of 26 °C. Detection was conducted at 280 nm, and the results were expressed as milligrams of standard equivalent per gram of dehydrated sample (mg/g).

#### 2.3.4. FTIR Spectroscopy Screening

The characterization of functional groups in cacao samples was conducted using Fourier transform infrared spectroscopy in attenuated total reflectance mode (FTIR-ATR). A total of 50 mg of freeze-dried cotyledons were analyzed using a Nicolet iS50 spectrophotometer (Thermo Scientific, Madison, WI, USA), with background correction performed through Omnic v9.2 software. Subsequently, the spectra were recorded at room temperature, averaging 50 scans per sample, over a spectral range of 4000–500 cm^−1^, with a resolution of 4 cm^−1^.

### 2.4. Statistical Processing

Statistical analyses and graphical representation of data pooled from two consecutive years of agromorphological characterization were performed using the R environment v4.4.3 [41], applying a combined univariate and multivariate analytical approach (Figure 2).

#### 2.4.1. Analysis of Frequencies and Genetic Components

Quantitative data were initially cleaned by detecting and excluding outliers using the *outliers* package v0.15 [42]. Subsequently, the variability of quantitative traits was described with the *summarytools* package v1.0.1 [43], calculating the mean, standard deviation (DevSt), extreme values (max and min), and coefficient of variation (CV). Frequency distributions were graphically represented with the *ggplot2* package v3.5.2 [44], employing histograms for quantitative descriptors and bar plots for qualitative ones.

Using the *variability* package v0.1.0 [45], which integrates the respective equations [1], the following genetic parameters were estimated: genotypic variance (GV) and phenotypic variance (PV), genotypic and phenotypic coefficients of variation (GCV and PCV), broad-sense heritability (H^2^), selection response expressed as genetic advance (GA), and genetic advance as a percentage of the mean (GAM).

#### 2.4.2. Correlation and Principal Component Analysis

The magnitude and direction of phenotypic associations among pairs of quantitative descriptors were assessed using Pearson’s correlation coefficient, calculated with the *Hmisc* package v5.2-3 [46]. To identify the quantitative descriptors contributing most to the total variability of the germplasm, a principal component analysis (PCA) was performed on the standardized data matrix (mean = 0; standard deviation = 1) using the *FactoMineR* v2.12 [47] and *Factoextra* v1.0.7 [48] packages. Eigenvalues and eigenvectors were computed for each principal component, and only those components with eigenvalues greater than 1 and a cumulative variance explaining at least 75% of the total variability were retained [49,50]. Finally, the first two principal components were represented in a biplot generated with the *ggplot2* package.

#### 2.4.3. Cluster Analysis and Genetic Distance

Hierarchical Cluster Analysis (HCA) was conducted using Euclidean distance and Ward’s minimum variance method (Ward.D2), implemented in the *cluster* package v2.1.8.1 [51]. The optimal number of clusters (K) was initially determined through the average silhouette index calculated with the *factoextra* package, and subsequently verified using the Gap Statistic method from the *cluster* package. Cluster stability was assessed by multiscale bootstrap resampling with the *pvclust* package v2.2-0 [52]. The graphical representation of the clustering was generated as a heatmap with associated dendrograms using the *ComplexHeatmap* v2.24.1 [53] and *dendextend* v1.19.1 [54] packages.

Genetic distance analysis was performed based on the Euclidean distance matrix and the cluster vector derived from the HCA. For this purpose, the customized function “cluster_distance” was used to compute the average inter- and intra-group distances.

Following the identification of phenotypic groups through HCA, assumptions of normality (Kolmogorov–Smirnov test) and homogeneity of variances (Bartlett’s test) were verified, considering a significance level of *p* ≥ 0.05. Differences among groups were evaluated using an unbalanced one-way analysis of variance (ANOVA), in which each accession was treated as a replicate within its respective phenotypic group, according to a fixed-effects linear model [55].

(3) $$Y_{ij}=\mu +\alpha_{i}+\varepsilon_{ij}$$

where $Y_{ij}$ represents the jth observation within the ith phenotypic group, μ denotes the overall mean of the descriptor, $\alpha_{i}$ corresponds to the fixed effect of the ith phenotypic group, and $\varepsilon_{ij}$ represents the independent random error.

Multiple comparisons were performed using Tukey’s HSD test at a 95% confidence level, implemented in the *agricolae* package v1.3-7 [56].

#### 2.4.4. Analysis of Promising Accessions

Promising accessions in terms of productivity were identified through using a bivariate linear mixed model under Bayesian inference, implemented in the *MCMCglmm* package [57] with Monte Carlo simulations through Markov chains and non-informative prior distributions [58]. Convergence was assessed with the *coda* v0.19.4 package [59], and the Bayesian Yield Stability Index (BYSI) was calculated as the minimum yield with a 90% probability of being exceeded [58], with uncertainty expressed through highest posterior density (HPD) intervals. The results were summarized in a biplot generated using *ggplot2*.

Differences among phytochemical parameters were determined using a one-way analysis of variance (ANOVA, *p* < 0.05), after verifying data normality with the Shapiro–Wilk test (*p* ≥ 0.05). Multiple comparisons were conducted using Tukey’s HSD test at a 95% confidence level with the *agricolae* package, and results were visualized using *ggplot2* and *tidyverse* v2.0.0 [60]. Additionally, CIELAB coordinates were graphically represented using *ggplot2* and *ggforce* v0.5.0 [61], while FTIR spectra were normalized and plotted using the *ChemoSpec* v6.3.0 [62] and *ggplot2* packages.

## 3. Results

### 3.1. Variation Patterns in Quantitative and Qualitative Descriptors

**Figure 3:**
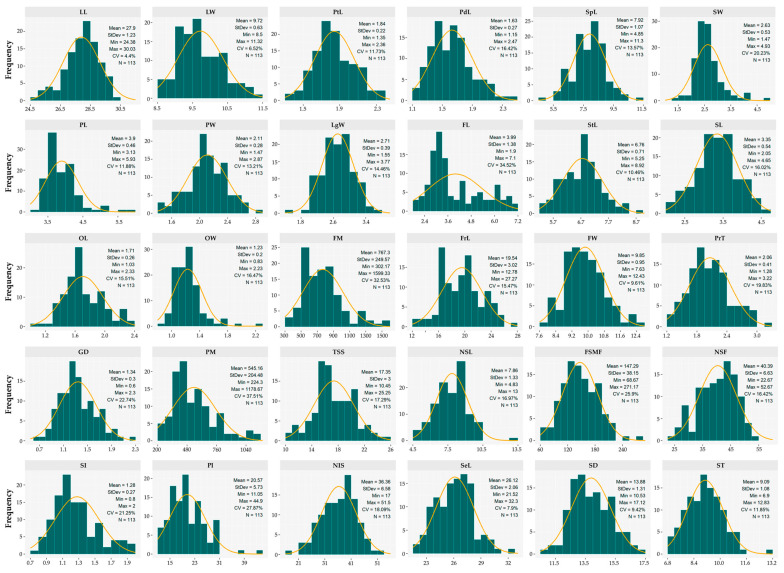
Distribution histograms of the germplasm for the quantitative descriptors. LL = Leaf length (cm); LW = Leaf width (cm); PtL = Petiole length (cm); PdL = Pedicel length (cm); SpL = Sepal length (mm); SW = Sepal width (mm); PL = Petal length (mm); PW = Petal width (mm); LgW = Ligule width (mm); FL = Filament length (mm); StL = Staminode length (mm); SL = Style length (mm); OL = Ovary length (mm); OW = Ovary width (mm); FM = Fruit mass (g); FrL = Fruit length (cm); FW = Fruit width (cm); PrT = Pericarp thickness (cm); GD = Groove depth (cm); PM = Pericarp mass (g); TSS = Total soluble solids (°Brix); NSL = Number of seeds per locule (Unit); FSMF = Fresh seed mass per fruit (g); NSF = Number of seeds per fruit (Unit); SI = Seed index (g); PI = Pod index (Unit); NIS = Number of intact seeds (Unit); SeL = Seed length (mm); SD = Seed diameter (mm); ST = Seed thickness (mm).

The quantitative assessment of 113 cacao accessions revealed substantial phenotypic diversity, supported by the values of CV, means, and ranges (Figure 3). The analysis of the pooled data from two evaluation campaigns showed moderate variability among foliar descriptors. LL ranged from 24.38 to 30.03 cm (CV = 4.4%), with a higher frequency of accessions occurring between 28.00 and 28.50 cm. LW varied between 8.50 and 11.32 cm (CV = 6.52%), with the highest frequency of accessions clustered in the 9.00–9.75 cm range.

Regarding floral traits, PdL ranged from 1.15 to 2.47 cm (CV = 16.42%), with the majority of accessions concentrated between 1.30 and 1.80 cm. SpL exhibited the highest frequency of accessions between 8.00 and 8.50 mm (CV = 13.57%). StL ranged from 5.25 to 8.92 mm (CV = 10.46%). Finally, OL within the evaluated germplasm fluctuated between 1.03 and 2.33 mm (CV = 15.51%), with the highest frequency occurring within the 1.60 to 1.70 mm interval.

Concerning fruit characteristics, FM exhibited a broad range of variation from 302.17 to 1599.33 g (CV = 32.53%), showing a higher frequency of accessions within the 500 to 900 g interval (Figure 3). FrL showed the highest frequency of accessions between 16 and 17 cm, whereas in FW ranged from 8.80 to 10.00 cm (CV = 9.61%). Fruits were primarily characterized by a PrT ranging from 1.28 to 3.22 cm (CV = 19.83%), with the highest proportion of accessions between 1.64 and 2.40 cm, and an average SH of 0.36 MPa (Figure S3).

The sweetness of the mucilage covering the cotyledons (TSS) up to 25.25 °Brix (CV = 17.29%) with the highest frequency of accessions concentrated between 15 and 17 °Brix. Likewise, most accessions produced between 32.5 and 50 seeds per fruit (NSF; CV = 16.42%) and an average of only 4.03 empty seeds (Figure S3). Regarding individual seed mass (SI), the highest frequency of accessions fell within the 1.10 to 1.20 g range, reaching up to 2 g (CV = 21.25%). These attributes are associated with larger seed dimensions, including SeL and ST, which ranged from 10.53 to 17.12 mm and from 6.90 to 12.83 mm, respectively. Additionally, PI values ranged between 11.05 and 44.90, with a mean of 20.57.

**Figure 4:**
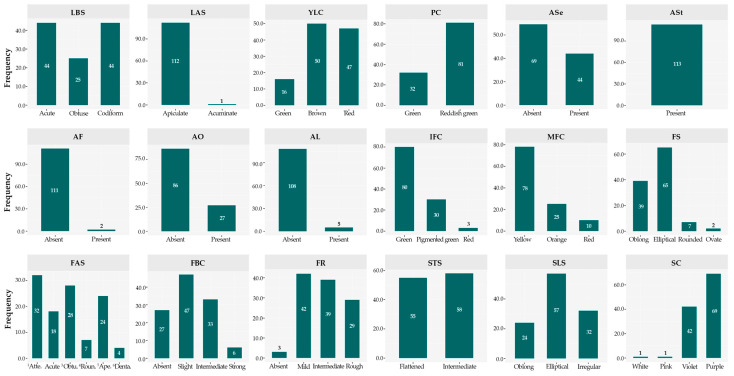
Frequency distribution of the germplasm for qualitative descriptors. LBS = Leaf base shape; LAS = Leaf apex shape; YLC = Young leaf color; PC = Pedicel color; ASe = Anthocyanin in sepals; ASt = Anthocyanin in staminodes; AF = Anthocyanin in filament; AO = Anthocyanin in ovary; AL = Anthocyanin in ligule; IFC = Immature fruit color; MFC = Mature fruit color; FS = Fruit shape; FAS = Fruit apex shape (^1^Attenuate, ^3^Obtuse, ^4^Rounded, ^5^Apezonate and ^6^Dentate); FBC = Fruit basal constriction; FR = Fruit roughness; STS = Seed transversal shape; SLS = Seed longitudinal shape; SC = Seed color.

**Figure 5:**
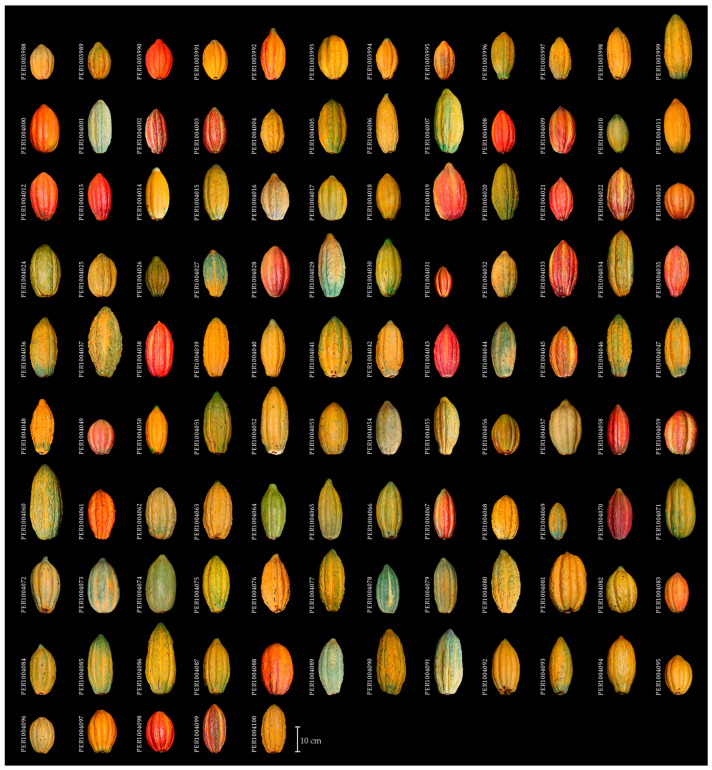
Phenotypic diversity among cacao accessions from the INIA Germplasm Bank, collected in the Amazonas region of Peru.

Qualitative characters (Table 1) were classified into distinct categories according to the phenotypic variability detected, adjusting the number of classes to the specificity of each morphotype. This approach enabled a systematic characterization of the diversity, revealing both frequent traits and others with uncommon occurrence within the evaluated set (Figure 4 and Figure 5). Among the 113 accessions analyzed, the apiculate LAS trait was predominant, being observed in 112 accessions. Regarding the LBS, an even distribution was observed between acute and codiform shapes (n = 44 each), while the obtuse shape was less frequent (n = 25). Concerning the YLC, brown (n = 50) predominated, followed by red (n = 47), both being more represented than green (n = 16).

In floral structures, most accessions exhibited a PC with a reddish green hue (n = 81), while a smaller number displayed a green hue (n = 32). Regarding anthocyanin presence, variation was observed across distinct floral organs within the collection: ASe (n = 44), AF (n = 2), AO (n = 27), and AL (n = 5). Conversely, ASt was consistently observed in all accessions (n = 113), suggesting potential genetic fixation of this trait.

For fruit descriptors, IFC was predominantly green (n = 80), followed by pigmented green (n = 30). In contrast, MFC was mainly yellow (n = 78), with lower frequencies of orange (n = 25) and red (n = 10) coloration. With respect to FS, elliptical (n = 65) and oblong (n = 39) forms were dominant, whereas rounded (n = 7) and ovate (n = 2) morphologies were uncommon. FR was mostly classified as mild (n = 42), intermediate (n = 39), or rough (n = 29).

Regarding seed traits, the STS was almost evenly distributed between flattened (n = 55) and intermediate (n = 58) categories, while the SLS was predominantly elliptical (n = 57). For SC, purple (n = 69) and violet (n = 42) were the most frequent, whereas white and pink were rare (n = 1 each), suggesting either recessive inheritance or low phenotypic expression of these color variants within the evaluated germplasm.

### 3.2. Estimation of Quantitative Genetic Parameters

Phenotypic characterization constitutes the foundation for assessing the diversity within a germplasm collection; however, its effectiveness is intrinsically conditioned by the degree of association between the observable phenotypic expressions and their underlying genetic basis (Table 2).

In the leaf descriptors, all traits exhibited low GCV and GAM values (<10%), indicating limited genetic variability and low potential for selection response. Furthermore, only LW and LL showed moderate broad-sense heritability estimates (H^2^ > 30%), suggesting the influence of transmissible genetic effects. In contrast, within the floral descriptors, several traits such as PdL, SW, and OW exhibited moderate GCV values (>10%), whereas FL stood out with the highest value (32.36%). For PCV, the traits SW, FL and SL showed elevated values (>20%). Overall, H^2^ was generally moderate to high (>30%) for most floral descriptors, except for SL, which recorded a markedly low value (1.55%). Moreover, the GAM exceeded 10% for ten of the eleven floral traits assessed, indicating a moderate to high genetic potential and suggesting an adequate capacity for response to selection.

For the fruit descriptors, FM and PM presented high values of GCV, PCV, and GAM (>20%), suggesting strong potential for genetic improvement through selection. Meanwhile, SH, NL, and TSS exhibited maximum H^2^ values (100%), indicating that the observed variation is completely attributable to stable genetic effects. Regarding seed characteristics, FSMF and NES showed high GCV (21.31% and 28.48%, respectively). Notably, FSMF exhibited both moderately high H^2^ (54.48%) and high GAM (32.41%), reflecting strong potential for selection response. Finally, PI and NES reached PCV values above 30%, highlighting a greater influence of environmental factors on their phenotypic expression.

### 3.3. Correlation and Principal Component Analysis

The Pearson correlation coefficient revealed distinct levels of association among the evaluated morphological structures (Table S2). Strong correlations were identified primarily among fruit and seed traits, with notable associations between FM and PM (0.95***), FW and PM (0.80***), NSL with NSF (0.87***), and NIS (0.89***), as well as between NSF and NIS (0.93***). Regarding moderate correlations, these were heterogeneously distributed across the analyzed morphometric structures, with a higher incidence in floral descriptors, where the correlation coefficient (r) ranged from 0.30 to 0.46. Overall, most traits exhibited weak correlations, suggesting that the quantitative descriptors tend to vary independently, reflecting the influence of distinct genetic control mechanisms and environmental modulation.

**Figure 6:**
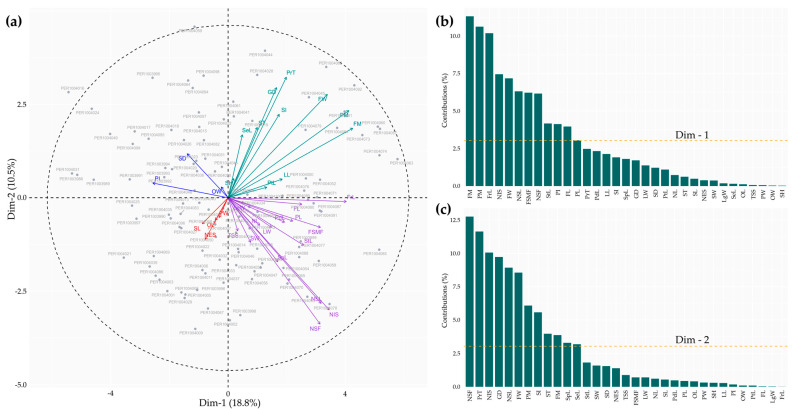
Principal component analysis. (**a**) Biplot of Dim-1 and Dim-2 illustrating the distribution of the 113 accessions and the projection of the 33 quantitative descriptors within a 95% confidence ellipse; (**b**,**c**) contribution of the descriptors to the formation of the first two principal dimensions. LL = Leaf length (cm); LW = Leaf width (cm); PtL = Petiole length (cm); PdL = Pedicel length (cm); SpL = Sepal length (mm); SW = Sepal width (mm); PL = Petal length (mm); PW = Petal width (mm); LgW = Ligule width (mm); FL = Filament length (mm); StL = Staminode length (mm); SL = Style length (mm); OL = Ovary length (mm); OW = Ovary width (mm); SH = Shell hardness (MPa); FM = Fruit mass (g); FrL = Fruit length (cm); FW = Fruit width (cm); PrT = Pericarp thickness (cm); GD = Groove depth (cm); PM = Pericarp mass (g); NL = Number of locules (Unit); TSS = Total soluble solids (°Brix); NSL = Number of seeds per locule (Unit); FSMF = Fresh seed mass per fruit (g); NSF = Number of seeds per fruit (Unit); SI = Seed index (g); PI = Pod index (Unit); NIS = Number of intact seeds (Unit); NES = Number of empty seeds (Unit); SeL = Seed length (mm); SD = Seed diameter (mm); ST = Seed thickness (mm).

The PCA enabled the identification of variation patterns and relationships among the 33 quantitative descriptors evaluated in the cacao accessions (Figure 6). The first twelve dimensions (eigenvalues > 1) together accounted for 77.67% of the total variance (Table S3). The first dimension, which explained 18.8% of the variation, was dominated by fruit descriptors, among which FM (11.31%), PM (10.62%), and FrL (10.18%) showed the highest positive contributions (Figure 6a,b, Table S3), reflecting their close association with fruit size and weight characteristics. The second dimension explained 10.5% of the variability and was mainly influenced by seed descriptors such as NSF (−0.66), NIS (−0.59), and NSL (−0.55), which contributed 12.76%, 10.05%, and 8.92%, respectively (Figure 6a–c, Table S3), to the formation of this dimension, associated with higher yield potential. Finally, the homogeneous distribution of accessions around the biplot center (Figure 6a) indicates a consistent level of heterogeneity within the studied germplasm.

### 3.4. Structural Organization of Germplasm

**Figure 7:**
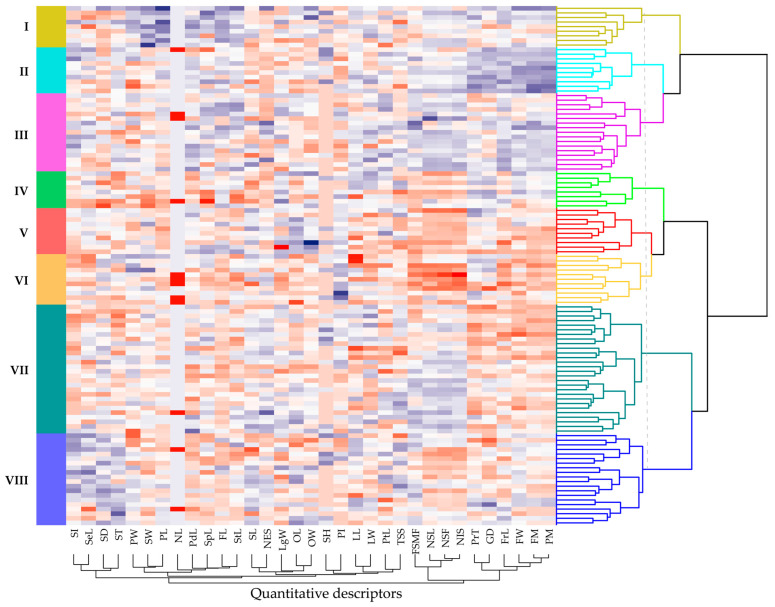
Dendrogram and heatmap generated through multivariate hierarchical clustering applied to 113 cacao accessions. Columns represent the different quantitative traits, where higher values are indicated by greater blue intensity and lower values by deeper red intensity. Quantitative descriptors are defined in Section Quantitative Descriptors of the paper.

The HCA based on quantitative descriptors identified eight phenotypic clusters (K = 8), with an average silhouette index of 0.20 (Figure S4), indicating an overall acceptable partitioning of variability within the germplasm collection. The eight clusters are displayed with distinct colors along the dendrogram rows (Figure 7). Moreover, the stability of the hierarchical structure, assessed through the multiscale bootstrap resampling, revealed that Clusters II and III exhibited high robustness, with approximately unbiased (AU) support values ≥ 95%, confirming their phenotypic consistency within the analyzed dataset.

A heterogeneous distribution of accessions was observed among clusters (Table S4), with Cluster VII comprising the largest proportion of accessions (24.78%), followed by Cluster VIII (17.70%) and III (15.04%), demonstrating a higher phenotypic representativeness within these groups. In contrast, Cluster IV exhibited the lowest representativeness (7.08%), whereas Clusters II and V displayed similar proportions (8.85%). Notably, according to the collection sites (Figure 1), accessions within each cluster originated from a broad range of altitudinal gradients, suggesting that the observed clustering pattern is primarily driven by intrinsic genetic variability, attributable to the genotype of each accession.

The data presented in Table 3 indicate that Cluster VIII exhibits the highest internal heterogeneity (intra-cluster distance = 7.54) and an average distance of 8.95, revealing substantial genetic variability among its accessions and positioning it as a promising source for broadening the parental diversity in breeding programs. Likewise, the greatest genetic divergences among clusters were observed between Cluster I–VII (10.03) and between Cluster I–VIII (9.85), suggesting that crosses among these groups could maximize heterosis. Conversely, the accessions belonging to Cluster II–III (7.41) and V–VII (7.46) exhibited the lowest genetic distances, indicating greater genetic similarity and, consequently, a reduced potential for complementary genetic combinations in their progenies.

### 3.5. Structural Analysis for Quantitative Descriptors

The clustering pattern revealed a heterogeneous distribution of the agromorphological attributes of interest among the different clusters, thus reflecting the structural diversity that exists among the accessions with agronomic potential (Table 4).

Regarding the floral descriptors, Cluster I exhibited the highest mean values in seven out of the eleven traits analyzed, notably SpL (8.88 mm), StL (7.58 mm), and PL (5.00 mm), indicating a more pronounced floral development. In contrast, Cluster V was distinguished by accessions with greater SL (3.71 mm) and OL (2.03 mm).

For the fruit descriptors, seven out of the nine traits with the highest mean values were recorded in Cluster II, highlighting FM (1267.08 g), PM (972.28 g), and fruit dimensions, with 23.52 cm for FrL and 11.28 cm for FW, suggesting enhanced structural and productive development within this group

Concerning seed descriptors, Cluster VIII exhibited the highest average SI (1.53 g), along with SD of 15.16 mm and ST of 10.20 mm. Conversely, accessions in Cluster III were characterized by their high prolificity, as evidenced by the highest mean values for NSL (9.09), NSF (46.02), and NIS (41.95), which resulted in a higher FSMF (180.4 g). Finally, Cluster II stood out for presenting the lowest mean PI value (17.63), indicating a better proportion of seed mass per fruit.

### 3.6. Structural Analysis for Qualitative Descriptors

**Figure 8:**
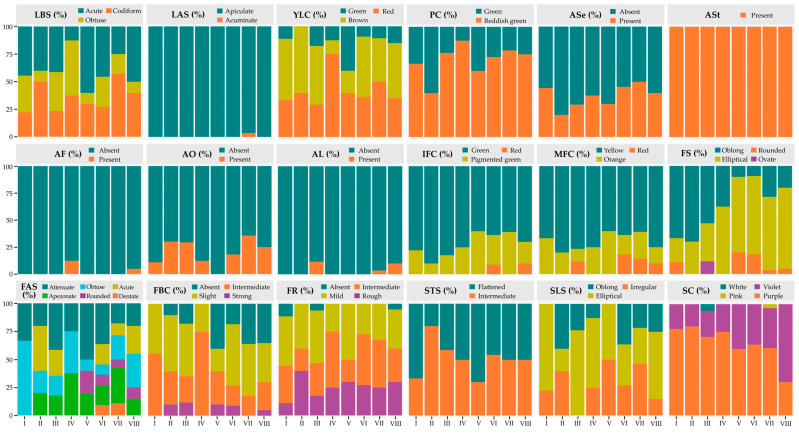
Frequency distribution of qualitative descriptors among clusters. LBS = Leaf base shape; LAS = Leaf apex shape; YLC = Young leaf color; PC = Pedicel color; ASe = Anthocyanin in sepals; ASt = Anthocyanin in staminodes; AF = Anthocyanin in filament; AO = Anthocyanin in ovary; AL = Anthocyanin in ligule; IFC = Immature fruit color; MFC = Mature fruit color; FS = Fruit shape; FAS = Fruit apex shape; FBC = Fruit basal constriction; FR = Fruit roughness; STS = Seed transversal shape; SLS = Seed longitudinal shape; SC = Seed color.

Frequency analysis revealed marked variation in morphological patterns among the accessions of the eight phenotypic clusters (Figure 8; Table S5). For the foliar descriptors, substantial variability was observed in LBS, with accessions from Cluster V predominantly exhibiting an acute base (60%), whereas Cluster IV showed a higher proportion with an obtuse base (50%). Conversely, Cluster VII was distinguished by a high frequency of accessions with a codiform shape (57.14%) and was the only group that exhibited acuminate apices (LAS; 3.57%).

With respect to floral descriptors, PC showed that only accessions in Cluster II displayed a high frequency of green color (60%), whereas in the remaining groups, reddish green predominated, exceeding 60%. The presence of anthocyanins in sepals was low across all groups recorded in less than 50% of accessions, while in staminodes (ASt), anthocyanins were consistently present in 100% of grouped accessions. Conversely, for the ovary, accessions in Cluster V were characterized by a complete absence of anthocyanins (100%).

Regarding fruit coloration, Cluster II and III predominantly exhibited a green hue for the IFC descriptor, with 90% and 82.35% of accessions, respectively. In contrast, only Clusters III, VI, VII, and VIII developed red coloration in variable proportions for the MFC descriptor, whereas yellow and orange hues were present across all clusters. Concerning FS, Clusters II and IV exclusively displayed oblong and elliptical fruits, whereas Cluster III included 11.76% of accessions with ovate fruits. With respect to the FR descriptor, this trait was most prominent in Cluster II, where 40% of the accessions exhibited a rough pericarp. In contrast, Cluster I, III, and VIII lacked this characteristic, with absence rates of 11.11%, 5.8%, and 5%, respectively.

In the seed descriptors, STS showed clear differences among clusters. In Cluster II, 80% of accessions exhibited an intermediate shape and 20% a flattened form; by contrast, Cluster V was characterized by a higher proportion of flattened seeds (70%). With respect to seed SC, only Cluster III contained accessions with white seeds (5.88%) and Cluster VII with pink seeds (3.57%), while violet and purple colorations predominated in varying proportions across the remaining groups (Table S5).

### 3.7. Selection of Promising Accessions

**Figure 9:**
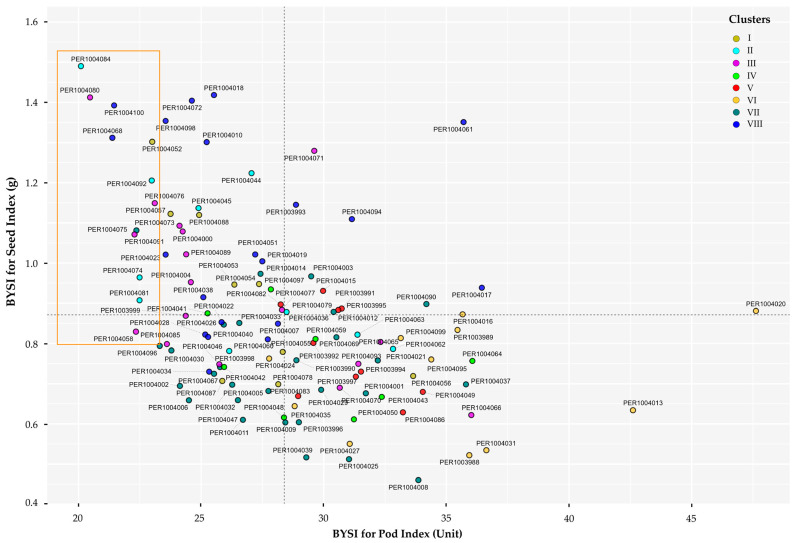
Bivariate distribution and multigroup selection diagram of promising plants in the germplasm of 113 cacao accessions. The orange rectangle highlights the promising accessions identified within the different clusters.

The scatter diagram (Figure 9, Table S6) illustrates the combined selection of accessions using the BYSI, simultaneously considering two discriminant descriptors closely linked to prolificity attributes due to their relation with traits associated with yield potential. Superior accessions were identified as those exceeding 0.8 g in seed index (SI) and presenting a pod index (PI) below 23.5, located in quadrants II and III of the Cartesian plane.

Based on the selection diagram, 12 accessions with high potential were identified (Table S6), representing 10.62% of the characterized germplasm and distributed across different districts in the province of Bagua and Utcubamba (Table S1). Among these, accessions PER1004084 and PER1004080 stood out, demonstrating a higher average dry seed mass (>1.4 g) and requiring only 21 fruits to yield 1 kg of dry seeds (PI), compared to accession PER1004076 (PI = 23.119). Conversely, PER1004018, although also exhibiting seeds with high dry mass, required more than 25 fruits to produce 1 kg of dry seeds. Furthermore, the desirable traits were distributed across different phenotypic clusters, indicating that yield potential is not confined to a single group but rather dispersed among the accessions within the evaluated germplasm collection.

Finally, the characterization of the germplasm under a permanent environment across two consecutive seasons revealed moderate to high repeatability values (r^ > 0.40) for both discriminant descriptors (Table S7). These results highlight that these traits possess intermediate to high heritability magnitude, supporting their reliability for characterization and selection in genetic improvement programs.

### 3.8. Phytochemical Profile of Selected Cacao Cotyledons

**Figure 10:**
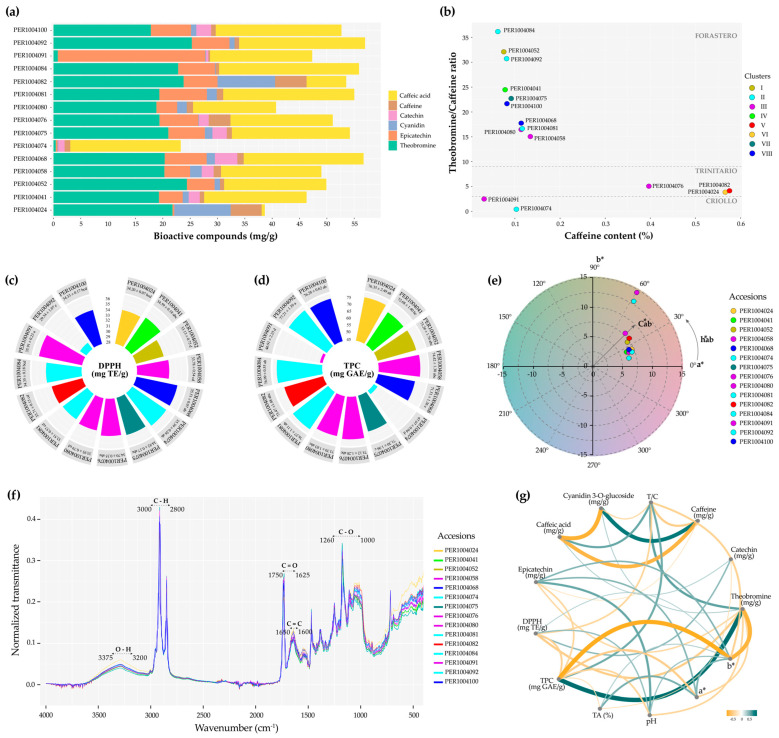
Phytochemical characterization of cacao cotyledons: (**a**) bioactive compound content; (**b**) classification of cacao accessions; (**c**,**d**) polyphenol and antioxidant contents among accessions, color-coded according to the phenotypic clusters defined in this study, where different letters indicate significant differences among clusters (Tukey’s HSD test; *p* ≤ 0.05); (**e**) colorimetric analysis of lyophilized cotyledons in the a* and b* plane; (**f**) FTIR analysis of functional groups; and (**g**) Pearson correlation network.

The analysis of bioactive compound content (Figure 10a and Table S8) revealed that among the 15 accessions evaluated, PER1004092 exhibited the highest theobromine content (25.34 ± 0.50 mg/g), whereas PER1004082 had the highest caffeine concentration (5.76 ± 0.22 mg/g). PER1004091 was notable for its epicatechin content (26.98 ± 0.26 mg/g), while catechin was absent in 7 of the 15 accessions studied.

Figure 10b illustrates a heterogeneous genetic structure, with two accessions (PER1004074 and PER1004091) clustering within the Criollo genetic group, whereas the majority (10 out of 15) group within the Forastero clade, encompassing accessions whose phenotypic profiles correspond to the distinct groups defined in this study.

The highest antioxidant and total polyphenol were detected in accessions PER1004091 (DPPH = 35.91 mg TE/g) and PER1004092 (TPC = 77.21 mg GAE/g), respectively (Figure 10c,d; Table S8). On the other hand, Figure 10e reveals a chromatic shift toward reddish–yellowish tones, as indicated by the clustering of lyophilized cotyledons within the positive quadrant of the a* and b* axes in the CIELAB space, albeit with varying intensities.

Figure 10f displays the normalized FTIR spectra of lyophilized cotyledons from 15 accessions, revealing a high degree of spectral similarity and the presence of characteristic bands associated with functional groups such as hydroxyl (O–H), hydrocarbons (C–H), carbonyls (C=O), aromatics and alkenes (C=C), esters, and carbohydrates (C–O). These results underscore the functional potential of the analyzed accessions as fine-flavor cacao.

The Pearson correlation network among phytochemical compounds (Figure 10g; Table S9) reflects significant associations, highlighting strong positive correlations between cyanidin 3-O-glucoside and caffeine (r = 0.86***), as well as between TPC and theobromine (r = 0.95***). Additional positive correlations were observed among epicatechin, color coordinates (a* and b*), and pH, as well as between TA and color. In contrast, strong negative correlations were detected between TPC and color b* (r = −0.92***), and between caffeic acid and caffeine (r = −0.83***).

## 4. Discussion

### 4.1. Ex Situ Germplasm Collection Management

The establishment of a germplasm bank of fine-flavor native cacao in the Amazon region of northeastern Peru represents a strategic initiative to safeguard genetic diversity and enhance its effective use in breeding programs aimed at benefiting future generations. This region, acknowledged as one of the centers of origin of cacao, harbors genotypes of high agronomic and commercial value, characterized by their potential for producing functional chocolates, their reduced cadmium uptake and translocation, and their high yield potential associated with significant disease tolerance [37,63]. The variability encompassed within these native Amazonian cacao genetic resources offers a valuable reservoir of adaptive alleles that can be harnessed to enhance crop productivity and resilience under changing climatic conditions.

In the Amazonas region, recent scientific advances have elucidated the intraspecific relationships of fine-flavor cacao population, revealing a considerable proportion of heterozygous genotypes and a reduced presence of homozygous individuals among accessions from the provinces of Bagua and Utcubamba [64]. This finding supports the high phenotypic diversity documented in the present germplasm collection, which is of is particularly relevant given that its integration with advanced biotechnological tools provides a promising framework for cacao genetic improvement in Peru. In this context, the incorporation of approaches such as CRISPR-Cas9 based genome editing approaches to discriminate between fine-flavor and bulk cacao, combined with the use of multi-omics platforms, could markedly accelerate the introgression of desirable phenotypic and sensory traits, thereby enhancing breeding efficiency [65,66,67]. Although the adoption of genomic approaches in developing countries continues to face technical and infrastructural constraints, sustained collaboration between local institutions and international research centers remains essential to overcome these challenges and ensure that scientific progress directly benefits smallholder farmers.

As part of strategies for the conservation and the sustainable utilization of terrestrial ecosystems within the framework of the Sustainable Development Goals (SDGs), an *ex situ* collection of fine-flavor cacao germplasm was successfully established in the Amazonas region, achieving a 100% survival rate due to the implementation of standardized grafting techniques and the strategic use of the IMC 67 clone as rootstock. This result not only confirms the effectiveness of the established protocol but also underscores the agronomic attributes of IMC 67, whose vigorous root system and tolerance to soil-borne pathogens likely enhanced nutrient uptake efficiency without significantly altering the physical or organoleptic characteristics of the scion fruits [68,69]. Despite its relatively narrow genetic base, the IMC 67 clone has consistently demonstrated performance and high efficiency across different regions of the country, establishing itself as a reliable rootstock for clonal propagation and the germplasm bank establishment. These findings reinforce its strategic importance in cacao conservation and genetic improvement programs in the Amazon region [68].

### 4.2. Phenotyping of Genetic Resources

Understanding the population structure and diversity within the center of origin of cacao is crucial for guiding conservation strategies and promoting the sustainable utilization of native varieties in production systems [70]. As a perennial, cross-pollinated crop with a diploid genome (2n = 20), cacao exhibits inherently high levels of heterozygosity [71]. This condition implies that crosses between two plants generate offspring with high genetic variability and a non-uniform distribution of traits, thereby ensuring evolutionary survival and providing opportunities for crop improvement [72,73].

The expression of both qualitative and quantitative traits in cacao is strongly conditioned by genotype–environment interactions [74]. In this regard, the Amazonas region stands out as a reservoir of diverse ecotypes, whose reproductive attributes reflect the combined effects of natural evolutionary processes and human-mediated selection [21]. Although conventional phenotyping is fundamental for defining cacao breeding goals, characterization based solely on external morphological traits has inherent limitations; while valuable for assessing existing diversity, it is inherently subjective and dependent on the evaluator’s perception [75]. Such constraints reduce its precision as an identification tool, underscoring the importance of integrating genotypic approaches to enhance the delineation and classification of germplasm collections.

Within the evaluated germplasm, leaf characterization allowed the identification of plants exhibiting a larger leaf area, attributed to both their dimensions and a morphology predominantly defined by an apiculate apex with an acute and codiform base. These plants also displayed young leaves with brownish to reddish pigmentation, a distinctive feature during the early stages of leaf development. This phenotypic variation is likely associated with a complex genetic architecture, resulting from the interaction between determinant genes and multiple loci with polygenic effects, which may underlie a multifactorial inheritance pattern linked to adaptive mechanisms and the expression of a higher diversity index [76,77]. Collectively, these findings align with recent studies demonstrating that the remarkable phenotypic variability of cacao arises from the cumulative effects of long-term natural selection and human management practices historically exerted across diverse Amazonian microenvironments [68,78].

Cauliflory represents the principal distinguishing feature of the cacao flowering pattern, characterized by the emergence of protandrous flowers directly from the trunk and main branches, forming structures known as floral cushions [74,79]. In the evaluated germplasm, the remarkable morphological variability at the floral level constitutes a highly valuable trait for characterization and conservation purposes. This diversity is influenced by the interaction between the genotype and the positional context of the flower on the plant, both of which determine its morphology and frequency of occurrence [80]. In this context, previous studies have demonstrated that anthocyanin pigmentation in floral organs such as the pedicel, staminodes, and ligule exhibits the highest discriminatory power among accessions within cacao germplasm collections [77,81].

In the analyzed germplasm, anthocyanins were consistently detected in the staminodes, with over 80 accessions exhibiting anthocyanins in the filament, ovary, and ligule. This pigmentation pattern is characteristic of materials originating exclusively the Upper Amazon region of Peru [30]. The occurrence of this coloration may be explained by the complex interaction between floral morphogenetic traits and the selective pressures imposed by specific pollinators [82]. Moreover, the observed agromorphological variation in the flower’s traits could be modulated or stabilized through the strategic use of rootstocks, given their substantial influence on the phenotypic expression of the scion. Such an effect could contribute to reducing variability in floral characteristics, thereby enhancing the stability of productive yield in certain accessions [80,83].

Fruits exhibited marked heterogeneity in both shape and coloration among accessions throughout their developmental and ripening stages. Notably, fruits with pericarp thickness stand out for presenting a lower incidence of *Phytophthora palmivora*, whereas rougher surfaces were linked to greater susceptibility to the pathogen [84]. Likewise, fruits characterized by an attenuated apex have been reported to exhibit reduced water accumulation and retention on their surface compared with fruits possessing an obtuse or rounded apex [85]. Such structural traits serve as valuable indicators for distinguishing accessions that are tolerant or susceptible to *Phytophthora* species, emphasizing the need to implement comprehensive conservation and characterization strategies aimed at broadening the genetic base of cacao and enhancing its agronomic performance [86,87]. Collectively, these findings underscore that the phenotypic diversity documented in the evaluated collection represents a strategic reservoir for developing resilient varieties through targeted breeding programs adapted to specific phytosanitary conditions.

For the quantitative descriptors, all fruit-related traits in the collection exhibited high heritability values (>60%) and genotypic coefficients of variation exceeding 10% in 6.6% of the evaluated descriptors, indicating that genetic factors predominantly govern the phenotypic expression of these traits. This finding suggests that the observed variability is largely determined by genetic factors, with lower environmental influence [88]. In this context, several accessions were distinguished by their superior mean values for fruit length (19.54 cm) and width (9.85 cm), which surpassed the overall means of 16.21 cm and 8.38 cm, respectively, reported for 140 native cacao accessions collected in the Loreto region of Peru [68].

The morphological variability of cacao seeds may be associated with the xenia effect, a phenomenon in which pollen from other trees influences traits such as almond size and coloration, resulting in fruits exhibiting either uniform or mixed grain coloration [89]. However, white seed coloration is typically characteristic of Criollo-type accessions and certain Trinitario hybrids, whereas purple pigmentation predominates in Forastero-type materials [74,90,91]. Additionally, the occurrence of oblong-shaped seeds has been linked to more uniform fermentation during postharvest processing compared with oval or elliptical seeds, thereby exerting a positive influence on flavor profile development [92].

Regarding the quantitative traits of the seeds, the evaluated collection exhibited an average pod index of 20.57, which falls within the optimal range for selecting trees that require fewer pods to produce 1 kg of dry seed mass, as they combine cotyledons with an individual mass exceeding 1 g [33]. This attribute is associated with a higher fat content and a lower shell proportion, factors that contribute positively to crop productivity and bean quality [93,94]. However, the number of seeds per fruit is largely determined by the number of ovules present in the ovary, as well as the effectiveness of the pollination process [95]. Therefore, the synergistic evaluation of the pod index with the seed index constitutes highly heritable and decisive parameters for identifying promising accessions with an emphasis on higher yield potential [18,93,96].

The distinctiveness of Peruvian fine-flavor cacao has been supported by genetic matching studies, which identified distinctive multilocus profiles among accessions collected in the Amazonas region, suggesting possible differentiation between genetic groups for the different provinces of the region [70]. In the present study, multivariate analyses revealed eight clearly defined phenotypic groups, reinforcing the hypothesis of a heritable genetic basis underlying the observed morphological variation. This structural pattern is consistent, with previous studies highlighting value of agromorphological traits as indirect tools for inferring genetic diversity patterns within cacao populations [30].

Recent studies conducted in the Amazonas and Loreto regions of northeastern Peru have identified five genetic groups across collections of 146 and 140 accessions, respectively, each exhibiting distinct morphological characteristics [6,68]. These findings suggest that the inclusion of accessions from different altitudinal gradients enhances the overall genetic diversity within the populations [70]. However, the morphological variability documented for the same clone across regions highlights the necessity of standardizing the phenotypic criteria employed and of further investigating whether such variations represent novel genetic lineages, subpopulations, or closely related clade [18]. In this context, it should be emphasized that membership in a phenotypic group does not necessarily indicate superior agronomic performance, underscoring the importance of evaluating each accession individually throughout its production cycle under diverse environmental conditions to ensure its appropriate conservation and utilization.

The concurrence of multiple germplasm accessions within the same cluster, based on their affinity across various descriptors, enables a comprehensive classification that facilitates the identification of agronomically relevant strategies [23]. Clusters III and II were notable for comprising phenotypically consistent accessions with high reproducibility, distinguished by their greater mean seed mass and lower pod index. These characteristics make them preferential candidates for intra-group selection and clonal propagation due to their superior productive efficiency. In contrast, groups characterized by fruits with favorable biometric proportions but smaller and fewer seeds represent suitable parental materials for inter-group hybridizations aimed at exploiting heterosis and the complementarity of quantitative traits [11]. Furthermore, groups comprising accessions with advantageous seed and fruit attributes should be prioritized as maternal parental sources, given their larger cotyledon size and lower pod index. Conversely, groups characterized by high fruit load and superior plant health could serve as paternal parents, considering prospective evaluations of postharvest quality to substantiate their potential in breeding programs [97,98].

### 4.3. Phytochemical Profile of Fine-Flavor Cacao

Cacao beans are a natural source of methylxanthines, phenolic compounds, and antioxidants, whose moderate consumption enhances physiological functions, reduces stress, and promotes overall well-being beyond their nutritional value. However, elevated levels of methylxanthines intensify bitterness and astringency, partially masking fruity, sweet, and caramel notes that consumers perceive differently according to their preferred sensory profile of cacao [99,100]. Moreover, the high postharvest stability, bioavailability, and rapid excretion of methylxanthines support their functional efficacy, as demonstrated by their direct correlation with metabolites in biological fluids [40,101]. This characteristic underscores the potential of the evaluated accessions as reliable sources of bioactive compounds, strategically positioning them within the global fine-flavor cocoa market.

The theobromine/caffeine ratio is a phytochemical marker traditionally used to genetically differentiate cacao into the Criollo, Trinitario, and Forastero groups. Criollo cocoas exhibit values < 2, Trinitario types range between 2–9, and Forastero types display values > 9 [102]. Ratios below 2 are typically associated with fine-flavor Criollo genotypes, whereas values exceeding 9 are linked to sensory profiles characteristic of bulk cacao [103]. Nevertheless, recent evidence, consistent with our findings, highlights the potential of Forastero genotypes for fine-flavor cacao production [104], thereby redefining the traditional paradigm that had previously confined this category exclusively to Criollo varieties.

Fine-flavor cacao is also characterized by high concentrations of catechin and epicatechin. In this context, recent studies conducted in the province of Utcubamba (Amazonas) reported of only 0.20 mg/g of catechin and 0.50 mg/g of epicatechin [37], values markedly lower than the 4.10 mg/g and 7.66 mg/g observed in the PER1004023 accession from the studied collection. Nonetheless, the fermentation process, drive by fungi such as *Candida* and *Aspergillus* [105], together with Maillard reactions and Strecker degradation occurring during drying and roasting, substantially reduces bioactive compounds while simultaneously promoting the formation of aromatic molecules through the interaction of carbonyl and nitrogenous compounds [92]. This balance underscores the importance of selecting accessions with a strong capacity to synthesize these metabolites.

FTIR analysis of cacao almonds revealed characteristic signals corresponding to phenols (3562–3322 cm^−1^, O–H) and aromatic compounds (2925–2854 cm^−1^, C–H). Consistent with our results, previous studies have reported the presence of these same functional groups in processed almonds [106,107], confirming the persistence of chemical groups associated with antioxidant capacity. Moreover, the peaks observed between 1750 and 1625 cm^−1^ in our spectra correspond to the characteristic stretching vibrations of carboxylic acids and aldehydes (C=O), fatty acids (C=C), and proteins (C=O), as similarly reported for Ecuadorian cocoa. The same study associates the signal at 1510 cm^−1^ with phenols [108], the 1726 cm^−1^ peak to ester carbonyl groups [109] and the signal at 3302 cm^−1^ with alcohols and amines present in compounds such as serotonin. accordance with our findings, recent evidence indicates that cacao concentrates key functional groups within the 4000–1500 cm^−1^ range, representing a significant contribution to the chemical characterization of fine-flavor cacao [110].

## 5. Conclusions

This study reports the successful establishment and comprehensive characterization of a fine-flavor cacao germplasm collection in the Amazonas region of Peru, whose sustained survival over nearly a decade demonstrates the remarkable adaptability and physiological compatibility between the selected rootstocks and scions.

The broad phenotypic variability and high heritability observed within this collection enabled its classification into eight clearly differentiated groups, consolidating it as a strategic and high-value resource for research focused on cacao genetic improvement. This potential is further supported by the significant differences detected in the phytochemical profiles of promising accessions, which exhibited elevated bioactive potential as determined through complementary HPLC and FTIR analyses. Collectively, these findings highlight the collection as a critical reservoir of agromorphological diversity, essential for the development of high-yielding and functionally superior genotypes, with direct implications for conservation and breeding programs.

While this study provides valuable insights into the phenotypic diversity of cacao, certain limitations should be acknowledged. The pronounced influence of the genotype by environment interaction on the expression of the evaluated descriptors underscores the importance of validating these findings across a broader range of agroecological contexts to identify consistent and stable patterns of variation. Similarly, expanding the geographical scope of sampling beyond the evaluated provinces could reveal additional reservoirs of adaptive variability. Addressing these limitations will not only enhance the selection of resilient and productive genotypes but also strengthen conservation strategies, thereby ensuring the sustainability and competitiveness of Amazonian cacao in the face of climate change challenges and the growing demand for differentiated, high-quality products.

## Figures and Tables

**Table 1:** Morphological descriptors evaluated in the characterization of cacao germplasm.

Structure	Descriptor	Acronym	Categorical Indicator
Leaf	Leaf base shape	LBS	Acute (1), Obtuse (2), Rounded (3) and Codiform (4)
	Leaf apex shape	LAS	Apiculate (1), Acuminate (3) and Caudate (5)
	Young leaf color	YLC	Green (1), Brown (3) and Red (5)
Flower	Pedicel color	PC	Green (1), Reddish green (2) and Reddish (3)
	Anthocyanin in sepals	ASe	Absent (1) and Present (2)
	Anthocyanin in staminodes	ASt	Absent (1) and Present (2)
	Anthocyanin in filament	AF	Absent (1) and Present (2)
	Anthocyanin in ovary	AO	Absent (1) and Present (2)
	Anthocyanin in ligule	AL	Absent (1) and Present (2)
Fruit	Immature fruit color	IFC	Green (1), Pigmented green (2) and Red (3)
	Mature fruit color	MFC	Yellow (1), Orange (2), Green (3) and Red (4)
	Fruit shape	FS	Oblong (1), Elliptical (2), Obovate (3), Rounded (4) and Ovate (5)
	Fruit apex shape	FAS	Attenuate (1), Acute (2), Obtuse (3), Rounded (4), Apezonate (5) and Dentate (6)
	Fruit basal constriction	FBC	Absent (0), Slight (1), Intermediate (2) and Strong (3)
	Fruit roughness	FR	Absent (0), Mild (1), Intermediate (3) and Rough (5)
Seed	Seed transversal shape	STS	Flattened (1), Intermediate (2) and Rounded (3)
	Seed longitudinal shape	SLS	Oblong (1), Elliptical (2), Ovate (3) and Irregular (4)
	Seed color	SC	White (1), Pink (2), Violet (3) and Purple (4)

**Table 2:** Estimation of genetic variation indicators in quantitative descriptors.

Estructure	Descriptor	$\bar{X}$	GV	PV	GCV (%)	PCV (%)	H^2^ (%)	GA	GAM (%)
Leaf	LL	27.90	0.60	1.51	2.78	4.40	39.91	1.01	3.62
	LW	9.72	0.17	0.40	4.22	6.52	41.83	0.55	5.62
	PtL	1.84	0.01	0.07	6.35	14.10	20.28	0.11	5.89
Flower	PdL	1.63	0.05	0.09	14.26	17.95	63.11	0.38	23.34
	SpL	7.92	0.96	1.27	12.36	14.21	75.59	1.75	22.13
	SW	2.63	0.22	0.28	17.71	20.27	76.30	0.84	31.86
	PL	3.90	0.16	0.23	10.40	12.36	70.82	0.70	18.03
	PW	2.11	0.04	0.08	9.08	13.21	47.26	0.27	12.86
	LgW	2.71	0.10	0.19	11.43	16.16	50.05	0.45	16.66
	FL	3.99	1.67	2.06	32.36	35.98	80.90	2.39	59.96
	StL	6.76	0.36	0.50	8.84	10.46	71.38	1.04	15.38
	SL	3.35	0.01	0.65	2.99	24.02	1.55	0.03	0.77
	OL	1.71	0.04	0.09	11.89	17.43	46.50	0.29	16.70
	OW	1.23	0.03	0.05	14.31	17.64	65.76	0.29	23.90
Fruit	SH	0.36	0.01	0.01	27.90	27.90	100.00	0.21	57.48
	FM	767.30	53,286.70	68,984.54	30.08	34.23	77.24	417.94	54.47
	FrL	19.54	7.29	10.56	13.81	16.62	69.04	4.62	23.65
	FW	9.85	0.67	1.05	8.32	10.38	64.19	1.35	13.73
	PrT	2.06	0.13	0.19	17.58	20.97	70.27	0.63	30.36
	GD	1.34	0.08	0.10	21.17	23.31	82.47	0.53	39.61
	PM	545.16	34,718.83	47,675.19	34.18	40.05	72.82	327.56	60.08
	NL	4.89	0.02	0.02	2.57	2.57	100.00	0.26	5.30
	TSS	17.35	1.51	1.51	7.09	7.09	100.00	2.53	14.60
Seed	NSL	7.86	0.67	2.60	10.43	20.52	25.81	0.86	10.91
	FSMF	147.29	985.50	1809.02	21.31	28.88	54.48	47.73	32.41
	NSF	40.39	24.52	57.81	12.26	18.82	42.42	6.64	16.45
	SI	1.28	0.03	0.11	13.86	25.51	29.55	0.20	15.52
	PI	20.57	1.60	64.09	6.15	38.93	2.50	0.41	2.00
	NIS	36.36	18.69	61.61	11.89	21.59	30.33	4.90	13.49
	NES	4.03	1.32	8.40	28.48	71.86	15.71	0.94	23.26
	SeL	26.12	2.35	5.14	5.87	8.68	45.78	2.14	8.18
	SD	13.88	0.86	2.16	6.67	10.59	39.74	1.20	8.67
	ST	9.09	0.28	1.82	5.79	14.83	15.25	0.42	4.66

Genotypic coefficients of variation (GCV) and phenotypic coefficients of variation (PCV): low (<10%), moderate (10–20%), high (>20%); broad-sense heritability (H^2^): low (<30%), moderate (30–60%), high (>60%); genetic advance as a percentage of the mean (GAM): low (<10%), moderate (10–20%), high (>20%). LL = Leaf length (cm); LW = Leaf width (cm); PtL = Petiole length (cm); PdL = Pedicel length (cm); SpL = Sepal length (mm); SW = Sepal width (mm); PL = Petal length (mm); PW = Petal width (mm); LgW = Ligule width (mm); FL = Filament length (mm); StL = Staminode length (mm); SL = Style length (mm); OL = Ovary length (mm); OW = Ovary width (mm); SH = Shell hardness (MPa); FM = Fruit mass (g); FrL = Fruit length (cm); FW = Fruit width (cm); PrT = Pericarp thickness (cm); GD = Groove depth (cm); PM = Pericarp mass (g); NL = Number of locules (Unit); TSS = Total soluble solids (°Brix); NSL = Number of seeds per locule (Unit); FSMF = Fresh seed mass per fruit (g); NSF = Number of seeds per fruit (Unit); SI = Seed index (g); PI = Pod index (Unit); NIS = Number of intact seeds (Unit); NES = Number of empty seeds (Unit); SeL = Seed length (mm); SD = Seed diameter (mm); ST = Seed thickness (mm).

**Table 3:** Genetic distances among clusters.

Cluster	C_I_	C_II_	C_III_	C_IV_	C_V_	C_VI_	C_VII_	C_VIII_	Mean Distance
C_I_	**7.21**	-	-	-	-	-	-	-	8.81
C_II_	8.02	**6.21**	-	-	-	-	-	-	7.97
C_III_	7.58	7.41	**6.46**	-	-	-	-	-	8.17
C_IV_	8.41	7.51	7.70	**7.17**	-	-	-	-	8.20
C_V_	9.51	7.51	8.57	8.07	**6.90**	-	-	-	8.21
C_VI_	8.30	7.79	7.59	8.10	8.24	**7.00**	-	-	8.21
C_VII_	10.03	8.91	9.23	8.48	7.46	8.17	**6.27**	-	8.70
C_VIII_	9.85	8.62	9.07	9.15	8.10	9.29	8.59	**7.54**	8.95

Matrix of intra-cluster (bold diagonal) and inter-cluster distances of cacao accessions, calculated based on 33 quantitative descriptors.

**Table 4:** Overall means and standard deviation (S.D.) of 33 quantitative agromorphological descriptors by phenotypic clusters.

Descriptors	Cluster I Mean ± S.D	Cluster II Mean ± S.D	Cluster III Mean ± S.D	Cluster IV Mean ± S.D	Cluster V Mean ± S.D	Cluster VI Mean ± S.D	Cluster VII Mean ± S.D	Cluster VIII Mean ± S.D
LL	28.28 ± 0.68 ab	28.46 ± 0.60 a	28.13 ± 0.71 ab	28.34 ± 1.45 ab	27.17 ± 1.05 ab	26.87 ± 1.71 b	27.78 ± 1.26 ab	28.19 ± 1.28 ab
LW	9.93 ± 0.58 ab	9.76 ± 0.65 ab	10.02 ± 0.63 ab	10.14 ± 0.60 a	9.29 ± 0.35 b	9.31 ± 0.55 b	9.75 ± 0.75 ab	9.60 ± 0.40 ab
PtL	1.83 ± 0.23 a	1.88 ± 0.16 a	2.00 ± 0.22 a	1.85 ± 0.11 a	1.82 ± 0.25 a	1.78 ± 0.14 a	1.77 ± 0.21 a	1.83 ± 0.24 a
PdL	1.91 ± 0.23 a	1.71 ± 0.21 abc	1.79 ± 0.27 ab	1.45 ± 0.19 c	1.73 ± 0.19 abc	1.55 ± 0.11 bc	1.59 ± 0.31 bc	1.45 ± 0.16 c
SpL	8.88 ± 1.39 a	7.98 ± 1.29 abc	8.50 ± 0.89 ab	6.99 ± 0.37 c	8.01 ± 1.04 abc	7.69 ± 0.84 abc	7.92 ± 0.78 abc	7.40 ± 0.82 bc
SW	3.69 ± 0.64 a	2.43 ± 0.26 bc	2.59 ± 0.28 bc	2.18 ± 0.52 c	2.73 ± 0.45 b	2.79 ± 0.66 b	2.60 ± 0.28 bc	2.38 ± 0.32 bc
PL	5.00 ± 0.57 a	3.98 ± 0.33 b	3.91 ± 0.33 bc	3.56 ± 0.20 cd	3.52 ± 0.19 d	3.83 ± 0.32 bcd	3.78 ± 0.26 bcd	3.86 ± 0.27 bcd
PW	2.40 ± 0.23 a	1.98 ± 0.33 b	2.09 ± 0.29 ab	1.98 ± 0.22 b	2.05 ± 0.19 b	2.26 ± 0.31 ab	2.08 ± 0.23 ab	2.10 ± 0.27 ab
LgW	2.63 ± 0.44 ab	2.66 ± 0.25 ab	2.79 ± 0.40 ab	3.08 ± 0.35 a	2.73 ± 0.57 ab	2.50 ± 0.37 b	2.76 ± 0.28 ab	2.57 ± 0.41 b
FL	6.21 ± 1.03 a	4.75 ± 1.02 b	4.65 ± 1.52 b	4.39 ± 0.97 bc	4.16 ± 1.35 bc	2.82 ± 0.53 d	3.22 ± 0.67 cd	3.53 ± 1.13 bcd
StL	7.58 ± 0.62 a	6.93 ± 0.47 abc	7.27 ± 0.69 ab	6.47 ± 0.86 c	6.61 ± 0.73 bc	6.42 ± 0.66 c	6.53 ± 0.53 bc	6.57 ± 0.56 bc
SL	3.25 ± 0.44 ab	3.00 ± 0.34 b	3.32 ± 0.61 ab	2.83 ± 0.32 b	3.71 ± 0.44 a	3.33 ± 0.54 ab	3.46 ± 0.40 ab	3.45 ± 0.67 ab
OL	1.72 ± 0.20 b	1.56 ± 0.16 b	1.70 ± 0.16 b	1.62 ± 0.28 b	2.03 ± 0.21 a	1.47 ± 0.20 b	1.73 ± 0.25 ab	1.76 ± 0.31 ab
OW	1.35 ± 0.31 ab	1.20 ± 0.15 ab	1.15 ± 0.13 b	1.32 ± 0.23 ab	1.42 ± 0.34 a	1.14 ± 0.09 b	1.19 ± 0.12 ab	1.22 ± 0.19 ab
SH	0.39 ± 0.12 ab	0.43 ± 0.13 a	0.31 ± 0.02 b	0.33 ± 0.07 ab	0.34 ± 0.08 ab	0.35 ± 0.08 ab	0.36 ± 0.09 ab	0.38 ± 0.12 ab
FM	852.33 ± 148.86 bc	1267.08 ± 195.55 a	958.86 ± 162.81 b	769.7 ± 73.47 cd	606.5 ± 116.97 de	543.5 ± 125.93 e	618.6 ± 123.27 de	726.9 ± 132.81 cde
FrL	21.00 ± 2.26 ab	23.52 ± 1.71 a	22.40 ± 2.18 a	19.05 ± 2.25 bc	17.01 ± 1.90 c	16.77 ± 2.65 c	18.34 ± 2.12 bc	19.15 ± 2.43 bc
FW	10.32 ± 1.00 b	11.28 ± 0.56 a	10.30 ± 0.76 b	10.22 ± 0.98 b	9.50 ± 0.73 bc	9.06 ± 0.55 c	9.11 ± 0.56 c	10.07 ± 0.55 b
PrT	2.15 ± 0.37 b	2.67 ± 0.30 a	2.05 ± 0.26 bc	2.31 ± 0.28 ab	2.13 ± 0.47 b	2.18 ± 0.39 b	1.71 ± 0.29 c	2.01 ± 0.29 bc
GD	1.30 ± 0.24 bc	1.81 ± 0.15 a	1.32 ± 0.22 bc	1.56 ± 0.27 ab	1.37 ± 0.21 bc	1.36 ± 0.32 bc	1.16 ± 0.19 c	1.27 ± 0.35 bc
PM	642.40 ± 139.25 b	972.28 ± 137.75 a	663.81 ± 125.82 b	600.78 ± 86.20 b	398.56 ± 96.74 c	407.16 ± 110.11 c	401.39 ± 85.58 c	515.21 ± 127.65 bc
NL	5.00 ± 0.01 a	4.90 ± 0.32 ab	4.88 ± 0.33 ab	4.88 ± 0.35 ab	5.00 ± 0.01 a	4.55 ± 0.52 b	4.96 ± 0.19 a	4.90 ± 0.31 ab
TSS	16.05 ± 2.95 a	16.81 ± 0.45 a	17.85 ± 2.13 a	16.81 ± 3.95 a	16.10 ± 3.60 a	16.18 ± 1.13 a	17.94 ± 3.07 a	18.45 ± 3.48 a
NSL	8.50 ± 0.73 a	8.43 ± 0.64 ab	9.09 ± 1.42 a	7.15 ± 1.21 c	6.42 ± 0.58 c	6.52 ± 1.37 c	8.43 ± 0.84 ab	7.19 ± 0.83 bc
FSMF	136.3 ± 18.68 bc	166.9 ± 28.72 ab	180.4 ± 35.64 a	117.3 ± 11.39 cd	111.8 ± 23.35 cd	97.4 ± 21.99 d	155.1 ± 33.74 ab	160.4 ± 28.27 ab
NSF	43.48 ± 3.42 a	41.92 ± 4.40 ab	46.02 ± 3.58 a	35.15 ± 3.97 cd	32.83 ± 2.76 cd	31.88 ± 6.35 d	45.18 ± 4.16 a	37.31 ± 4.21 bc
SI	1.34 ± 0.26 abc	1.44 ± 0.28 ab	1.36 ± 0.25 abc	1.14 ± 0.14 c	1.18 ± 0.14 bc	1.11 ± 0.17 c	1.11 ± 0.18 c	1.53 ± 0.29 a
PI	18.68 ± 3.71 b	17.63 ± 5.05 b	18.06 ± 5.27 b	21.87 ± 4.45 b	23.49 ± 2.18 ab	29.18 ± 7.32 a	20.19 ± 4.30 b	18.82 ± 5.00 b
NIS	39.72 ± 4.73 a	39.78 ± 4.66 a	41.95 ± 4.61 a	33.04 ± 3.64 bc	29.15 ± 3.23 bc	27.44 ± 6.35 c	39.89 ± 3.73 a	33.27 ± 4.55 b
NES	3.76 ± 2.93 ab	2.14 ± 0.80 b	4.07 ± 1.62 ab	2.11 ± 0.85 b	3.68 ± 1.33 ab	4.44 ± 1.85 ab	5.29 ± 2.29 a	4.04 ± 1.70 ab
SeL	25.19 ± 2.12 ab	26.68 ± 2.11 ab	26.37 ± 1.75 ab	26.00 ± 1.91 ab	26.57 ± 1.11 ab	24.66 ± 1.93 b	25.64 ± 1.66 ab	27.37 ± 2.61 a
SD	13.37 ± 1.56 b	12.96 ± 0.74 b	13.32 ± 0.82 b	12.95 ± 1.81 b	13.59 ± 0.83 b	14.19 ± 1.15 ab	14.04 ± 1.05 ab	15.16 ± 1.16 a
ST	9.22 ± 1.03 abc	9.60 ± 0.55 ab	9.21 ± 0.90 abc	7.98 ± 0.92 d	8.82 ± 0.57 bcd	8.88 ± 1.01 bcd	8.49 ± 0.83 cd	10.20 ± 1.01 a

Mean values ± standard deviation, followed by different letters, indicate significant differences among clusters (Tukey’s HSD test; *p* ≤ 0.05). LL = Leaf length (cm); LW = Leaf width (cm); PtL = Petiole length (cm); PdL = Pedicel length (cm); SpL = Sepal length (mm); SW = Sepal width (mm); PL = Petal length (mm); PW = Petal width (mm); LgW = Ligule width (mm); FL = Filament length (mm); StL = Staminode length (mm); SL = Style length (mm); OL = Ovary length (mm); OW = Ovary width (mm); SH = Shell hardness (MPa); FM = Fruit mass (g); FrL = Fruit length (cm); FW =Fruit width (cm); PrT = Pericarp thickness (cm); GD = Groove depth (cm); PM = Pericarp mass (g); NL = Number of locules (Unit); TSS = Total soluble solids (°Brix); NSL = Number of seeds per locule (Unit); FSMF = Fresh seed mass per fruit (g); NSF = Number of seeds per fruit (Unit); SI = Seed index (g); PI = Pod index (Unit); NIS = Number of intact seeds (Unit); NES = Number of empty seeds (Unit); SeL = Seed length (mm); SD = Seed diameter (mm); ST = Seed thickness (mm).

## Data Availability

Data are contained within the article and Supplementary Materials.

## Supplementary Materials

The following supporting information can be downloaded at: https://www.mdpi.com/article/10.3390/plants14223536/s1, Figure S1: Germplasm collection of *Theobroma cacao* L. Figure S2: Hydrometeorological diagram of the studied cropping seasons. Figure S3: Distribution histograms of germplasm based on quantitative descriptors. Figure S4: Optimal number of clusters determined using the silhouette method. Table S1: Collection points. Table S2: Association analysis among quantitative descriptors. Table S3: PCA and eigenvalues of the first twelve dimensions derived from 33 quantitative descriptors in 113 Cacao Accessions. Table S4: Eight phenotypic clusters of cacao based on quantitative descriptors. Table S5: Percentage distribution of frequencies for qualitative descriptors. Table S6: BYSI estimation with the 95% highest posterior density interval for agromorphological yield descriptors in 113 cacao accessions. Table S7: Estimation of repeatability and variance components associated with seed and pod index in 113 cacao accessions from Amazonas, Peru. Table S8: Characterization of phytochemical compounds in cacao cotyledons. Table S9: Pearson correlation values for phytochemical compounds in cacao cotyledons.
