# Supplementary material for: Exploring the Agromorphological Profiles of the Cacao (Theobroma cacao L.) Collection from the INIA Germplasm Bank in the Amazonas Region, Peru
Source: Plants (Basel). 2025 Nov 19;14(22):3536. doi: 10.3390/plants14223536 (PMC12655995; doi:10.3390/plants14223536)
Supplement: Supplementary file 1 [file plants-14-03536-s001.zip › Supplementary Figures.pdf]

plants

MDPI

Article

Exploring the Agromorphological Profiles of the Cacao (*Theobroma cacao* L.) Collection from the INIA Germplasm Bank in the Amazonas Region, Peru

José Jesús Tejada-Alvarado [1,*], Nuri Carito Vilca-Valqui [1,*], Luis Alberto Montenegro-Acuña [1], Jhimy Andy Parco-Quinchori [2] and Elizabeth Fernandez [2]

1 Estación Experimental Agraria Amazonas, Dirección de Recursos Genéticos y Biotecnología, Instituto Nacional de Innovación Agraria (INIA), km 3.5 (Carretera Aeropuerto), Chachapoyas 01001, Perú; montenegro9824@gmail.com (L.A.M.-A.)

2 Centro Experimental La Molina, Dirección de Recursos Genéticos y Biotecnología, Instituto Nacional de Innovación Agraria (INIA), Av. La Molina 1981, Lima 15024, Perú; jhmparco@gmail.com (J.A.P.-Q.); efernandezh@inia.gob.pe (E.F)

* Correspondence: tejada.01634@gmail.com (J.J.T.-A.); nvilca@inia.gob.pe (N.C.V.-V.)

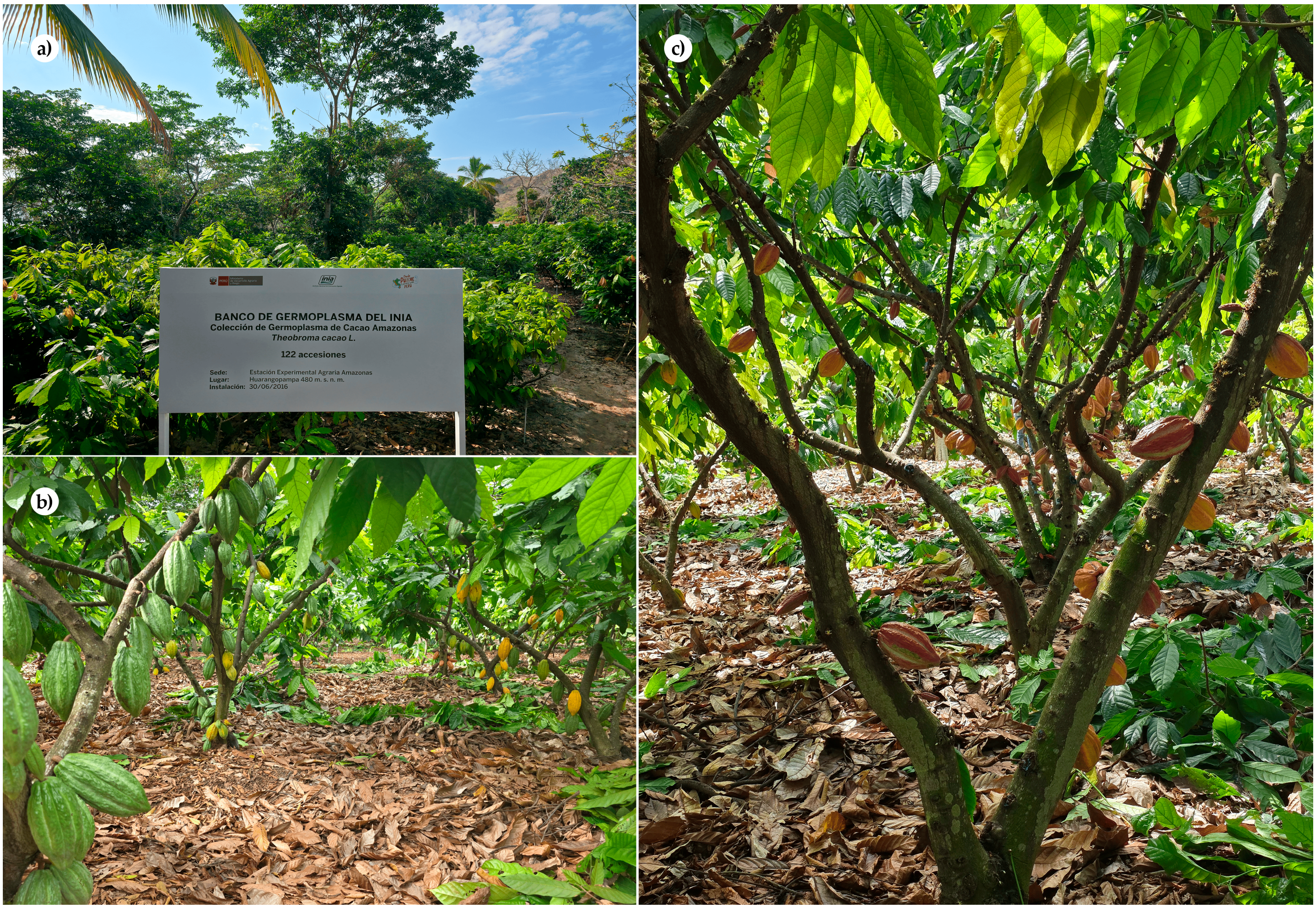

Figure S1. Germplasm collection of *Theobroma cacao* L. **a)** Labeling of the germplasm, highlighting the installation date and the composition of the collection, which comprises 122 accessions, of which 113 are of national origin and 9 of international provenance (data not shown). **b–c)** Details of the distribution and successful establishment of cacao trees bearing developing fruits.

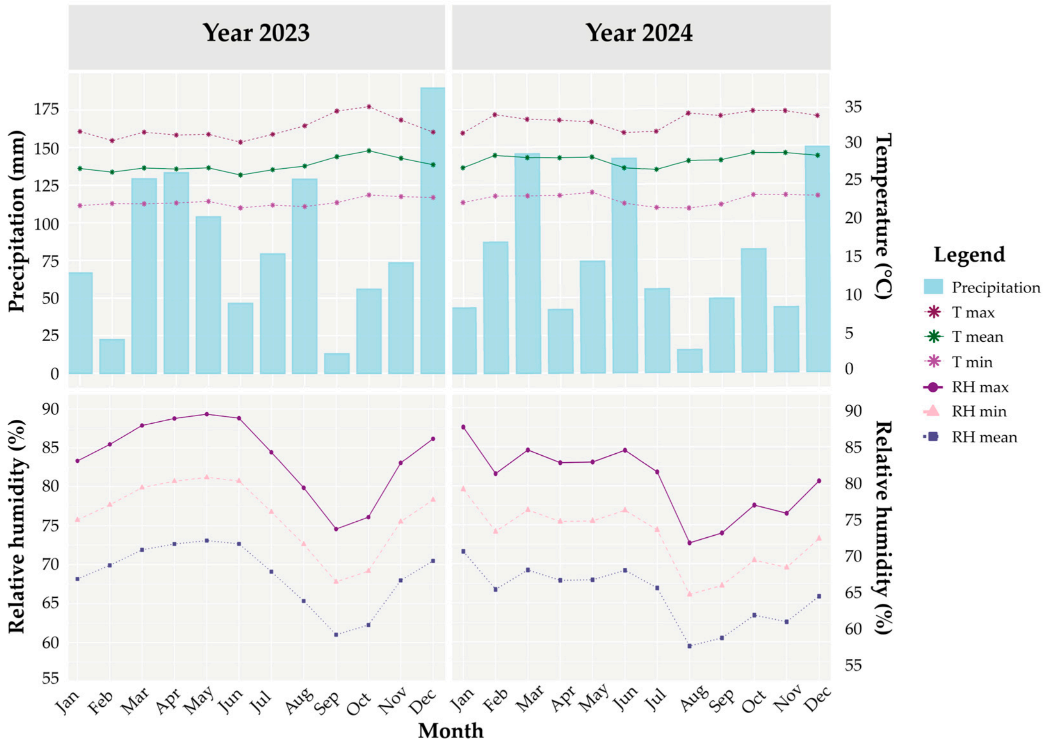

Figure S2. Hydrometeorological diagram of the studied cropping seasons.

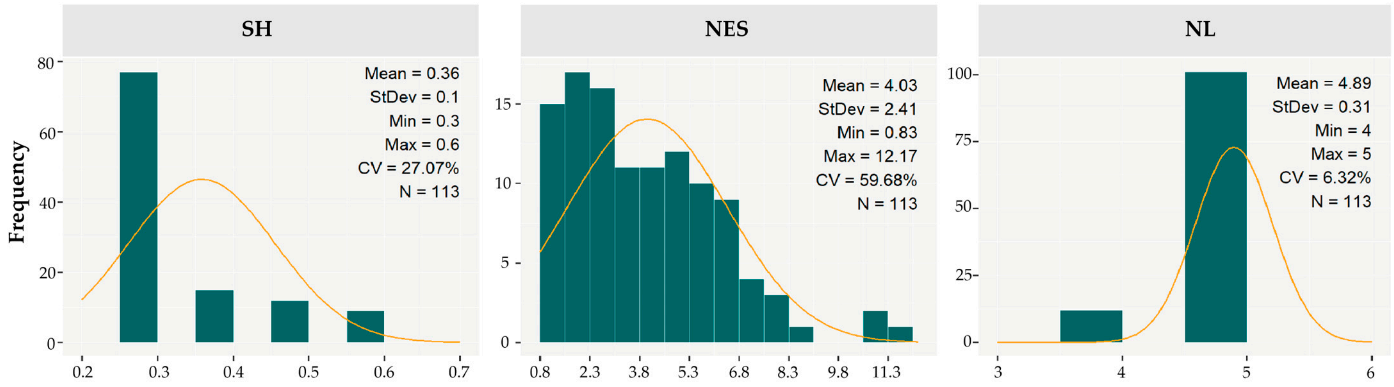

Figure S3. Distribution histograms of germplasm based on quantitative descriptors. SH = Shell hardness (MPa); NES = Number of empty seeds (Unit); NL = Number of locules (Unit).

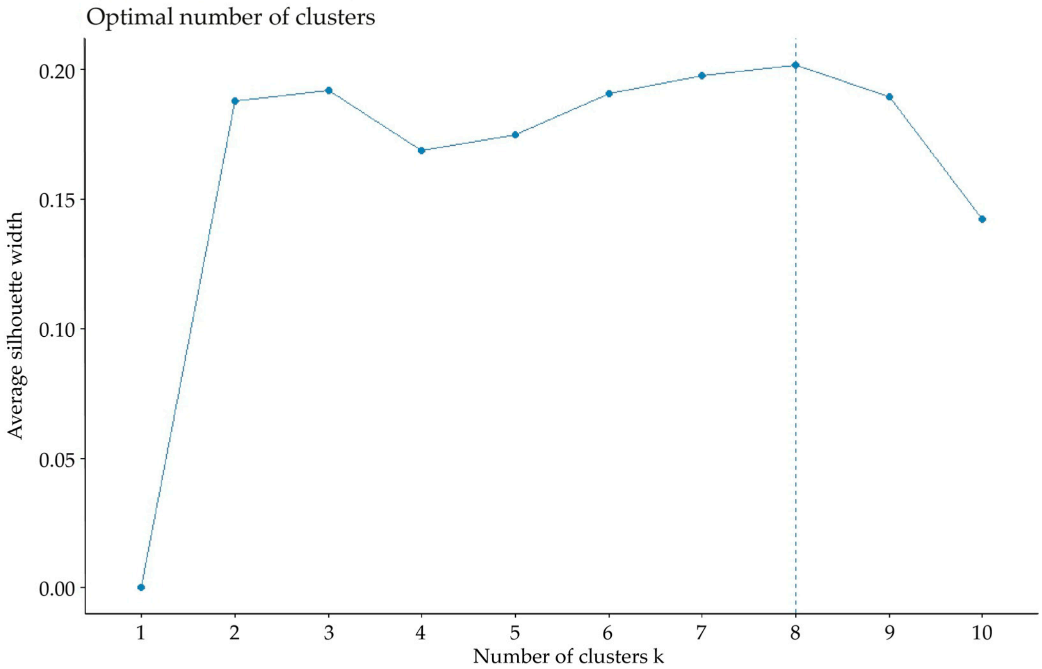

Figure S4. Optimal number of clusters determined using the silhouette method.
